# Bis(*μ*-4-amino-3,5-dimethyl-4*H*-1,2,4-triazole-*κ* ^2^ *N* ^1^:*N* ^2^)bis­(dibromidozinc)

**DOI:** 10.1107/S1600536811028789

**Published:** 2011-07-30

**Authors:** Xia Zhu, Ying Guo, Jian-Gang Li, Yao Wu

**Affiliations:** a Science College, Civil Aviation University of China, Tianjin 300300, People’s Republic of China

## Abstract

The centrosymmetric dimeric title complex, [Zn_2_Br_4_(C_4_H_8_N_4_)_2_], is isotypic with its [Zn_2_Cl_4_(C_4_H_8_N_4_)_2_], [Zn_2_I_4_(C_4_H_8_N_4_)_2_] and [Co_2_Cl_4_(C_4_H_8_N_4_)_2_] analogues. The zinc atom is bonded to two N atoms belonging to triazole bridging rings and to two terminal bromide ligands, in a geometry close to tetra­hedral. Weak N—H⋯Br hydrogen bonds, with the amine functions as donor groups, are observed in the crystal structure, forming a three-dimensional supra­molecular network.

## Related literature

For background to transition metal complexes of 1,2,4-triazole derivatives, see: Liu *et al.* (1999 ▶). For the isotypic [Zn_2_Cl_4_(C_4_H_8_N_4_)_2_], [Zn_2_I_4_(C_4_H_8_N_4_)_2_] and [Co_2_Cl_4_(C_4_H_8_N_4_)_2_] analogues, see: Lavrenova *et al.* (1992 ▶); Zhang *et al.* (2011 ▶); Gong *et al.* (2009 ▶). For other related structures, see: Liu *et al.* (2003 ▶); Zhao *et al.* (2002 ▶); Yi *et al.* (2004 ▶); Zhang *et al.* (2007 ▶).
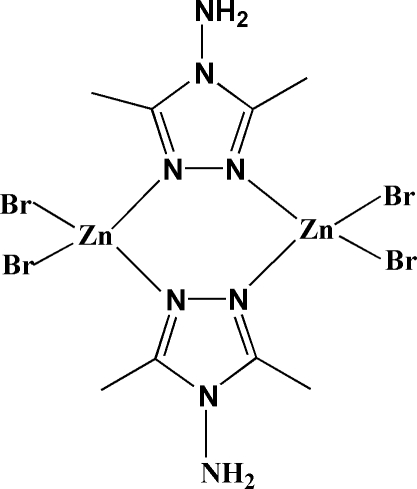


## Experimental

### 

#### Crystal data

[Zn_2_Br_4_(C_4_H_8_N_4_)_2_]
*M* *_r_* = 674.67
Monoclinic, 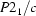

*a* = 7.0344 (17) Å
*b* = 12.629 (3) Å
*c* = 11.456 (3) Å
β = 99.951 (6)°
*V* = 1002.4 (4) Å^3^
*Z* = 2
Mo *K*α radiation
μ = 10.37 mm^−1^
*T* = 293 K
0.48 × 0.20 × 0.16 mm

#### Data collection

Rigaku Mercury CCD diffractometer
Absorption correction: multi-scan (*REQAB*; Jacobson, 1998 ▶) *T*_min_ = 0.083, *T*_max_ = 0.288
9580 measured reflections
1833 independent reflections
1517 reflections with *I* > 2σ(*I*)
*R*_int_ = 0.054

#### Refinement

*R*[*F* ^2^ > 2σ(*F* ^2^)] = 0.052
*wR*(*F* ^2^) = 0.148
*S* = 1.05
1833 reflections
110 parameters
2 restraints
H atoms treated by a mixture of independent and constrained refinement
Δρ_max_ = 0.69 e Å^−3^
Δρ_min_ = −0.97 e Å^−3^

### 

Data collection: *CrystalClear* (Rigaku, 2005 ▶); cell refinement: *CrystalClear*; data reduction: *CrystalClear*; program(s) used to solve structure: *SHELXS97* (Sheldrick, 2008 ▶); program(s) used to refine structure: *SHELXL97* (Sheldrick, 2008 ▶); molecular graphics: *SHELXTL* (Sheldrick, 2008 ▶); software used to prepare material for publication: *SHELXTL*.

## Supplementary Material

Crystal structure: contains datablock(s) I, global. DOI: 10.1107/S1600536811028789/bh2370sup1.cif

Structure factors: contains datablock(s) I. DOI: 10.1107/S1600536811028789/bh2370Isup2.hkl

Additional supplementary materials:  crystallographic information; 3D view; checkCIF report

## Figures and Tables

**Table:** 

Zn1—N1	2.027 (6)
Zn1—N2^i^	2.025 (6)
Zn1—Br1	2.3523 (12)
Zn1—Br2	2.3625 (12)

**Table:** 

N2^i^—Zn1—N1	107.5 (2)
N2^i^—Zn1—Br1	109.56 (16)
N1—Zn1—Br1	107.83 (17)
N2^i^—Zn1—Br2	109.48 (16)
N1—Zn1—Br2	108.79 (17)
Br1—Zn1—Br2	113.53 (5)

Symmetry code: (i) 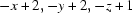.

**Table 2:** Hydrogen-bond geometry (Å, °)

*D*—H⋯*A*	*D*—H	H⋯*A*	*D*⋯*A*	*D*—H⋯*A*
N4—H4*D*⋯Br1^ii^	0.85 (2)	2.80 (7)	3.428 (7)	132 (8)
N4—H4*E*⋯Br2^iii^	0.86 (2)	2.93 (4)	3.748 (8)	161 (8)

Symmetry codes: (ii) 
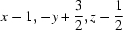
; (iii) 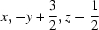.

**Fig. 1.**
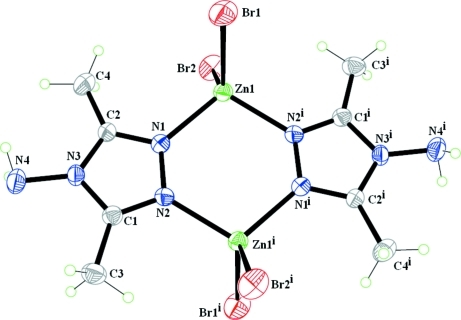
View of the title complex.

**Fig. 2.**
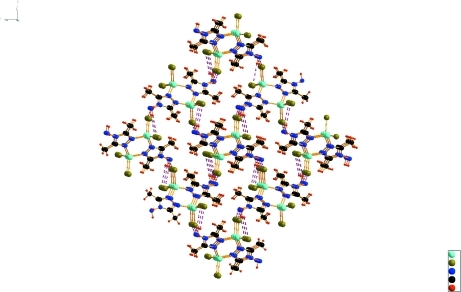
The crystal structure of the title complex.

## supplementary crystallographic information

### Comment

Transition metal complexes bridged by 1,2,4-triazole group can produce interesting structures and specific properties. Many attempts have been made to synthesize a variety of complexes with paramagnetic centers by using such ligands, and their structures and magnetic properties have been characterized (Liu *et al.*, 1999). For 4-amino-3,5-dimethyl-1,2,4-triazole (admt), several Cu^II^ (Liu *et al.*, 2003), Co^II^, Ni^II^ (Zhao *et al.*, 2002; Gong *et al.*, 2009), and Cd^II^ compounds (Yi *et al.*, 2004) were synthesized. However, to the best of our knowledge, only two Zn^II^-admt compounds, [Zn_2_(admt)_2_Cl_4_] and [Zn_2_(admt)_2_I_4_] were synthesized (Lavrenova *et al.*, 1992; Zhang *et al.*, 2011). Here, we report the preparation and crystal structure of a dimeric Zn^II^ complex of formula [Zn_2_(admt)_2_Br_4_].

The structure of the title compound is made up of neutral dimeric metallacycles. The title compound is isostructural to analogous complexes which were previously reported: [Zn_2_(admt)_2_Cl_4_], [Zn_2_(admt)_2_I_4_] and [Co_2_(admt)_2_Cl_4_] (Lavrenova *et al.*, 1992; Zhang *et al.*, 2011; Gong *et al.*, 2009). In each dimeric metallacycle, as shown in Fig. 1, two Zn^II^ centers are connected by two admt ligands, resulting in a discrete Zn_2_(admt)_2_ 6-membered metallacycle, which represents the smallest closed cyclic structure with a 1:1 metal-to-ligand ratio. Two triazole rings are coplanar. Each Zn^II^ center is four-coordinated with two N donors of two admt ligands [Zn1—N1: 2.027 (6) Å; Zn1—N2^i^ (symmetry code i: 2-*x*, 2-*y*, 1-*z*): 2.025 (6) Å] and two Br^-^ anions ligands [Zn1—Br1: 2.3523 (12) Å; Zn1—Br2: 2.3625 (12) Å], forming a distorted tetrahedral geometry. The Zn—N(triazole) bond lengths in the title compound are consistent with values in other Zn-triazole complexes (Zhang *et al.*, 2007, 2011; Lavrenova *et al.*, 1992). The N—Zn—N, N—Zn—Br and Br—Zn—Br bond angles in the title compound are in the range of 107.5 (2)° to 113.53 (5)°, near to the ideal tetrahedral value of *ca* 109.5°. The ligand admt is a 4-substituted 1,2,4-triazole and exhibits in the title compound the *κ*^2^*N*^1^:*N*^2^ bidentate bridging coordination mode. Two admt ligands bridge two Zn^II^ ions to form a dimer with a Zn···Zn separation of 3.7781 (6) Å. For a 4-substituted 1,2,4-triazole, by blocking the N4 donor position through substitution, only the N1 monodentate (Zhang *et al.*, 2007) and N1,N2-bidentate coordination modes are possible.

There are weak hydrogen bonding interactions between the H atoms of the amine NH_2_ groups and the Br^-^ anions of adjacent dimers (N4—Br1^ii^ = 3.428 (7) Å, N4—Br2^iii^ = 3.748 (8) Å; symmetry codes: ii = 1-*x*, 3/2-*y*, *z*-1/2; iii = *x*, 3/2-*y*, *z*-1/2). The adjacent dimers are held together by N—H···Br hydrogen bonds to form a three-dimensional supramolecular network (Fig. 2). No obvious π···π stacking interactions between the triazole rings are observed in the crystal structure.

### Experimental

To a solution of admtrz in EtOH was added one equivalent of ZnBr_2_ (aqueous solution) under stirring at room temperature. Then, the reaction mixture was filtered and colorless crystals suitable for structure determination were isolated by slow evaporation of the solvent at room temperature after a couple of weeks.

### Refinement

H atoms of the methyl groups were placed in idealized positions and refined as riding, with C—H distances of 0.96 Å and *U*_iso_(H) = 1.5*U*_eq_(parent C). H atoms bonded to N4 were located in a difference map and refined with N—H distances restrained to 0.85 (2) Å, and with *U*_iso_(H) = 1.2*U*_eq_(N4).

### Crystal data

**Table:**

[Zn_2_Br_4_(C_4_H_8_N_4_)_2_]	*F*(000) = 640
*M**_r_* = 674.67	*D*_x_ = 2.235 Mg m^−^^3^
Monoclinic, *P*2_1_/*c*	Mo *K*α radiation, λ = 0.71070 Å
Hall symbol: -P 2ybc	Cell parameters from 2994 reflections
*a* = 7.0344 (17) Å	θ = 3.2–25.4°
*b* = 12.629 (3) Å	µ = 10.37 mm^−^^1^
*c* = 11.456 (3) Å	*T* = 293 K
β = 99.951 (6)°	Block, colourless
*V* = 1002.4 (4) Å^3^	0.48 × 0.20 × 0.16 mm
*Z* = 2	

### Data collection

**Table:**

Rigaku Mercury CCD diffractometer	1833 independent reflections
Radiation source: fine-focus sealed tube	1517 reflections with *I* > 2σ(*I*)
graphite	*R*_int_ = 0.054
Detector resolution: 14.63 pixels mm^-1^	θ_max_ = 25.3°, θ_min_ = 3.2°
ω scans	*h* = −8→8
Absorption correction: multi-scan (REQAB;Jacobson, 1998)	*k* = −13→15
*T*_min_ = 0.083, *T*_max_ = 0.288	*l* = −13→13
9580 measured reflections	

### Refinement

**Table:**

Refinement on *F*^2^	Primary atom site location: structure-invariant direct methods
Least-squares matrix: full	Secondary atom site location: difference Fourier map
*R*[*F*^2^ > 2σ(*F*^2^)] = 0.052	Hydrogen site location: inferred from neighbouring sites
*wR*(*F*^2^) = 0.148	H atoms treated by a mixture of independent and constrained refinement
*S* = 1.05	*w* = 1/[σ^2^(*F*_o_^2^) + (0.084*P*)^2^ + 1.6395*P*] where *P* = (*F*_o_^2^ + 2*F*_c_^2^)/3
1833 reflections	(Δ/σ)_max_ < 0.001
110 parameters	Δρ_max_ = 0.69 e Å^−^^3^
2 restraints	Δρ_min_ = −0.97 e Å^−^^3^
0 constraints	

### Fractional atomic coordinates and isotropic or equivalent isotropic displacement parameters (Å^2^)

**Table:**

	*x*	*y*	*z*	*U*_iso_*/*U*_eq_	
Zn1	0.91275 (11)	0.89737 (6)	0.59841 (7)	0.0379 (3)	
Br1	1.05816 (13)	0.72906 (7)	0.61240 (10)	0.0726 (4)	
Br2	0.66056 (12)	0.91347 (7)	0.70931 (9)	0.0638 (3)	
N1	0.8048 (8)	0.9248 (4)	0.4253 (5)	0.0393 (13)	
N2	0.8846 (8)	0.9908 (4)	0.3485 (5)	0.0372 (13)	
N3	0.6514 (8)	0.9028 (4)	0.2457 (5)	0.0386 (13)	
N4	0.5184 (11)	0.8698 (6)	0.1475 (6)	0.0541 (17)	
H4D	0.408 (7)	0.881 (7)	0.166 (8)	0.06 (3)*	
H4E	0.520 (13)	0.803 (2)	0.161 (8)	0.06 (3)*	
C1	0.7888 (10)	0.9765 (5)	0.2415 (6)	0.0381 (15)	
C2	0.6619 (10)	0.8725 (5)	0.3605 (6)	0.0398 (16)	
C3	0.8174 (12)	1.0313 (6)	0.1318 (6)	0.0527 (19)	
H3A	0.6998	1.0660	0.0969	0.079*	
H3B	0.8526	0.9805	0.0768	0.079*	
H3C	0.9184	1.0828	0.1503	0.079*	
C4	0.5307 (13)	0.7968 (7)	0.4037 (8)	0.063 (2)	
H4A	0.6048	0.7423	0.4489	0.094*	
H4B	0.4473	0.7657	0.3374	0.094*	
H4C	0.4544	0.8332	0.4528	0.094*	

### Atomic displacement parameters (Å^2^)

**Table:**

	*U*^11^	*U*^22^	*U*^33^	*U*^12^	*U*^13^	*U*^23^
Zn1	0.0342 (5)	0.0373 (5)	0.0419 (5)	−0.0009 (3)	0.0058 (4)	0.0031 (3)
Br1	0.0570 (6)	0.0457 (6)	0.1112 (9)	0.0139 (4)	0.0036 (5)	0.0004 (4)
Br2	0.0528 (5)	0.0687 (6)	0.0770 (7)	−0.0005 (4)	0.0308 (5)	−0.0099 (4)
N1	0.038 (3)	0.039 (3)	0.040 (3)	−0.005 (2)	0.004 (3)	0.003 (2)
N2	0.037 (3)	0.038 (3)	0.035 (3)	−0.003 (2)	0.005 (3)	0.005 (2)
N3	0.036 (3)	0.039 (3)	0.037 (3)	−0.003 (2)	−0.002 (3)	−0.004 (2)
N4	0.053 (4)	0.058 (5)	0.048 (4)	−0.017 (4)	0.001 (3)	−0.008 (3)
C1	0.040 (4)	0.037 (4)	0.038 (4)	−0.002 (3)	0.009 (3)	0.000 (3)
C2	0.039 (3)	0.041 (4)	0.038 (4)	−0.009 (3)	0.003 (3)	0.003 (3)
C3	0.070 (5)	0.048 (5)	0.041 (4)	−0.001 (4)	0.013 (4)	0.004 (3)
C4	0.072 (6)	0.054 (5)	0.061 (5)	−0.025 (4)	0.005 (4)	0.002 (4)

### Geometric parameters (Å, °)

**Table:**

Zn1—N1	2.027 (6)	N4—H4D	0.85 (2)
Zn1—N2^i^	2.025 (6)	N4—H4E	0.86 (2)
Zn1—Br1	2.3523 (12)	C1—C3	1.478 (10)
Zn1—Br2	2.3625 (12)	C2—C4	1.472 (10)
N1—C2	1.320 (8)	C3—H3A	0.9600
N1—N2	1.398 (8)	C3—H3B	0.9600
N2—C1	1.305 (9)	C3—H3C	0.9600
N2—Zn1^i^	2.025 (6)	C4—H4A	0.9600
N3—C1	1.349 (9)	C4—H4B	0.9600
N3—C2	1.359 (9)	C4—H4C	0.9600
N3—N4	1.397 (9)		
			
N2^i^—Zn1—N1	107.5 (2)	N2—C1—N3	108.6 (6)
N2^i^—Zn1—Br1	109.56 (16)	N2—C1—C3	127.6 (6)
N1—Zn1—Br1	107.83 (17)	N3—C1—C3	123.8 (6)
N2^i^—Zn1—Br2	109.48 (16)	N1—C2—N3	108.2 (6)
N1—Zn1—Br2	108.79 (17)	N1—C2—C4	126.7 (6)
Br1—Zn1—Br2	113.53 (5)	N3—C2—C4	125.1 (6)
C2—N1—N2	107.1 (5)	C1—C3—H3A	109.5
C2—N1—Zn1	125.8 (5)	C1—C3—H3B	109.5
N2—N1—Zn1	126.5 (4)	H3A—C3—H3B	109.5
C1—N2—N1	108.1 (5)	C1—C3—H3C	109.5
C1—N2—Zn1^i^	126.9 (5)	H3A—C3—H3C	109.5
N1—N2—Zn1^i^	124.4 (4)	H3B—C3—H3C	109.5
C1—N3—C2	108.0 (5)	C2—C4—H4A	109.5
C1—N3—N4	124.2 (6)	C2—C4—H4B	109.5
C2—N3—N4	127.7 (6)	H4A—C4—H4B	109.5
N3—N4—H4D	105 (6)	C2—C4—H4C	109.5
N3—N4—H4E	100 (6)	H4A—C4—H4C	109.5
H4D—N4—H4E	96 (8)	H4B—C4—H4C	109.5
			
N2^i^—Zn1—N1—C2	−176.0 (6)	Zn1^i^—N2—C1—C3	6.4 (11)
Br1—Zn1—N1—C2	66.0 (6)	C2—N3—C1—N2	1.2 (8)
Br2—Zn1—N1—C2	−57.5 (6)	N4—N3—C1—N2	178.3 (7)
N2^i^—Zn1—N1—N2	14.3 (7)	C2—N3—C1—C3	−177.7 (7)
Br1—Zn1—N1—N2	−103.7 (5)	N4—N3—C1—C3	−0.7 (11)
Br2—Zn1—N1—N2	132.7 (5)	N2—N1—C2—N3	0.6 (7)
C2—N1—N2—C1	0.1 (7)	Zn1—N1—C2—N3	−170.8 (5)
Zn1—N1—N2—C1	171.5 (5)	N2—N1—C2—C4	−177.4 (8)
C2—N1—N2—Zn1^i^	172.1 (5)	Zn1—N1—C2—C4	11.2 (11)
Zn1—N1—N2—Zn1^i^	−16.6 (8)	C1—N3—C2—N1	−1.1 (8)
N1—N2—C1—N3	−0.8 (8)	N4—N3—C2—N1	−178.1 (7)
Zn1^i^—N2—C1—N3	−172.6 (4)	C1—N3—C2—C4	176.9 (8)
N1—N2—C1—C3	178.1 (7)	N4—N3—C2—C4	0.0 (12)

Symmetry codes: (i) −*x*+2, −*y*+2, −*z*+1.

### Hydrogen-bond geometry (Å, °)

**Table:**

*D*—H···*A*	*D*—H	H···*A*	*D*···*A*	*D*—H···*A*
N4—H4D···Br1^ii^	0.85 (2)	2.80 (7)	3.428 (7)	132 (8)
N4—H4E···Br2^iii^	0.86 (2)	2.93 (4)	3.748 (8)	161 (8)

Symmetry codes: (ii) *x*−1, −*y*+3/2, *z*−1/2; (iii) *x*, −*y*+3/2, *z*−1/2.
